# Quantitative three-dimensional fracture mapping reveals subtype-specific morphology of acetabular roof column and wall fractures

**DOI:** 10.3389/fsurg.2026.1879706

**Published:** 2026-06-29

**Authors:** Zihan Liu, Zihang Zhao, Xi Hou, Changyong Ma, Zhiyong Hou, Lianxin Song, Ruipeng Zhang

**Affiliations:** 1 Department of Orthopaedic Surgery, Third Hospital of Hebei Medical University, Shijiazhuang, China; 2 Hebei Medical University, Shijiazhuang, China

**Keywords:** acetabular fracture, acetabular roof, fracture mapping, fracture morphology, preoperative planning, three-column classification, three-dimensional reconstruction

## Abstract

**Introduction:**

Acetabular roof column and wall fractures (A3 injuries) are uncommon injuries involving the acetabular roof, but their subtype-specific three-dimensional morphology remains incompletely quantified.

**Methods:**

This multicenter retrospective study characterized the fracture-line distribution and geometric features of A3.1–A3.3 injuries using standardized CT-based three-dimensional reconstruction and fracture mapping. Among 3,248 patients with acetabular fractures screened at nine level-I trauma centers between January 2017 and December 2022, 89 had A3 injuries, and 46 surgically treated patients with adequate CT data were included. CT data were reconstructed in Mimics and processed in 3-Matic for fragment separation, virtual reduction, fracture-line tracing, surface-area measurement, displacement assessment, and angular analysis. Fracture trajectories were registered to a standardized left-sided hemipelvic template to generate subtype-specific fracture maps and an overall heat map.

**Results:**

A3.1 fractures showed shorter three-dimensional perifragment fracture-boundary length but greater fragment displacement and angular change, whereas A3.2 and A3.3 fractures demonstrated longer cranial trajectories and larger extra-fossa surface involvement. No statistically significant difference in intra-fossa surface area was detected among subtypes. Classification agreement was substantial, and quantitative measurements showed excellent reliability.

**Discussion:**

Quantitative three-dimensional mapping identified distinct morphologic patterns among A3 roof injuries. Rotational displacement of a localized roof fragment was a key morphology-based concern in A3.1 injuries, whereas A3.2 and A3.3 injuries showed broader cranial extension and greater extra-fossa involvement that may warrant further investigation in relation to fixation coverage and clinical outcomes. Because the cohort included only surgically treated patients with adequate CT data, these findings should be interpreted primarily in the context of more displaced or complex A3 injuries and may not be generalizable to minimally displaced or nonoperatively managed injuries.

## 1 Introduction

Acetabular fractures that involve the acetabular roof are clinically important because this region carries the peak joint load and is critical for postoperative hip congruity. Within the three-column classification, the roof is further described as the roof column and roof wall, and isolated injury patterns in this zone have been summarized as a distinct subgroup in multicenter series. However, these fractures remain uncommon and heterogeneous, and practical evidence to guide surgical decision-making is still limited (1–3).

For roof column/wall injuries, treatment success depends on restoring the articular surface and stabilizing fragments that may be small, thin, and prone to rotational malreduction. Standard classification systems are essential for communication, but they do not quantify key morphologic features such as fragment displacement, rotation, and intra-fossa involvement. Even when a classification shows acceptable reproducibility, surgeons still need objective, measurable descriptors to support preoperative planning and to enable meaningful comparisons across centers and techniques (2, 4).

Three-dimensional (3D) fracture mapping offers a way to move from descriptive labels to pattern-based morphology, by reducing individual fractures on a template and superimposing fracture lines to visualize frequent zones and trajectories. Mapping studies in acetabular fracture subtypes have shown that fracture lines cluster in repeatable regions, and endopelvic zoning concepts further provide an anatomic “roadmap” that is aligned with contemporary intrapelvic approaches. These data suggest that a dedicated 3D mapping analysis focused on roof column/wall injuries could clarify typical fracture morphology and support morphology-informed preoperative assessment (4–7).

At the same time, modern 3D workflows (virtual reduction planning, 3D printing, and augmented reality) are increasingly used in complex acetabular surgery, but their value depends on reproducible measurements and clear operational definitions (8–10).

Therefore, we conducted a retrospective, multicenter study of surgically treated acetabular roof column/wall fractures to characterize fracture morphology using 3D mapping and heatmap visualization, and quantify clinically relevant morphologic metrics—including fragment displacement distance, articular/non-articular surface involvement, rotational angle, and post-reduction 3D perifragment fracture-boundary length—using a standardized 3D measurement protocol with reliability assessment.

## 2 Materials and methods

### 2.1 Study design and ethics

This multicenter retrospective study reviewed consecutive patients with acetabular fractures treated at nine level-I trauma centers between January 2017 and December 2022. The study was approved by the Institutional Review Board of the Third Hospital of Hebei Medical University (approval number: W2025-120-1). The requirement for informed consent was waived by the ethics committee owing to the retrospective design and the use of de-identified patient data.

### 2.2 Fracture classification

Fractures were classified according to the three-column classification system. In this system, A3 injuries refer to acetabular roof column or wall fractures. A3.1 represents roof wall fractures, A3.2 represents roof column fractures, and A3.3 represents complex roof column fractures with more extensive cranial or column involvement. All classifications were performed using preoperative CT images and three-dimensional reconstructions.

### 2.3 Eligibility criteria

Patients were considered for inclusion if they met all of the following: acetabular fracture treated during the study period; A3 acetabular roof column or wall fracture (A3.1–A3.3) on preoperative CT;

CT data adequate for 3D reconstruction. Exclusion criteria were: prior hip surgery, non-operative management, osteoporosis or pathological fracture, restricted hip range of motion before injury, age <18 years, or inadequate CT quality for 3D reconstruction. Cases unsuitable for contralateral mirroring, such as bilateral acetabular fractures or marked contralateral pelvic deformity, were considered ineligible.

### 2.4 CT processing and 3D reconstruction

Preoperative pelvic CT DICOM data were imported into Mimics 20.0 (Materialise, Leuven, Belgium) for bone segmentation and 3D model generation. The 3D models were then imported into 3-Matic 12.0 (Materialise, Leuven, Belgium) for fragment separation, virtual reduction, fracture-line tracing, and quantitative measurements. CT studies with incomplete coverage of the pelvis/acetabulum, marked motion/metal artifacts, or image quality insufficient for reliable segmentation and fragment delineation were excluded (thin-slice CT, typically ≤2 mm, was required for reconstruction).

### 2.5 Main fragment definition and virtual reduction

For each case, fracture subtype was first determined using the complete preoperative CT dataset and three-dimensional reconstruction according to the three-column classification criteria. The main fragment was then operationally defined for quantitative measurement as the largest reconstructable displaced fragment involving the acetabular roof and best representing the principal A3 fracture morphology. Depending on the fracture pattern, this fragment could represent a roof-wall fragment or a roof-column fragment. When more than one roof-related fragment was present, priority was given to the fragment with greater involvement of the superior acetabular roof/dome rather than peripheral iliac extension alone. Small free osteochondral fragments, marginal impaction fragments, and minor comminuted cortical fragments were not selected as the main fragment unless they were continuous with the dominant roof fragment. Borderline cases, including cases with two fragments of similar size or similar roof involvement, were reviewed jointly by the two observers and adjudicated by a senior acetabular surgeon.

In 3-Matic 12.0, the contralateral hemipelvis was mirrored to the injured side using the Mirror function and used as a reduction template. Template transparency was adjusted to facilitate fragment alignment. Fracture fragments were reduced by manual translations and rotations in multiple views and then refined using rigid registration. The reduced configuration was used as the reference for fracture mapping and measurements.

### 2.6 Fracture mapping and heat map generation

After virtual reduction, fracture lines were traced on the reduced acetabular surface. A standard left hemipelvic template was selected. For standardization on a common left-sided template, fracture lines from right-sided cases were mirrored to the left side before superimposition. Each patient's reduced acetabulum was then aligned to the template using scaling and rigid registration. To depict subtype-specific morphology, fracture lines were grouped by subtype (A3.1–A3.3) and superimposed separately on the same template. Fracture lines from all patients were then superimposed on the template to generate an overall composite fracture map, and a frequency heat map was generated to visualize the distribution of fracture lines. Because the number of fracture lines within each subtype was limited, subtype-level frequency heat maps were not generated; subtype results are presented as superimposed fracture-line plots.

### 2.7 Definition of injury energy

Injury mechanisms were grouped as high-energy (traffic accident, fall from height, crush injury) and low-energy (slip/trip fall).

### 2.8 Measurement definitions

All measurements were performed in 3-Matic 12.0 and Mimics 20.0.

#### 2.8.1 Fragment displacement (mm)

Three landmarks were placed on the main fragment along the long axis of the fracture line (two endpoints and one midpoint). Displacement was defined as the Euclidean distance between the landmark positions before and after virtual reduction. The mean of the three distances was used as the displacement value.

Surface areas (mm^2^). Using the Mark function, the main fragment surface was divided into the portion within the acetabular fossa (intra-fossa region) and the remaining portion outside the fossa (extra-fossa region). Surface areas of both regions were recorded.

#### 2.8.2 Angular change (degrees)

Angular change was used to describe the rotational displacement of the main roof fragment before virtual reduction. After three-dimensional reconstruction, fracture fragments were separated and displayed in different colors. The principal roof fragment (roof-column or roof-wall fragment, depending on the fracture pattern) was identified on the injured side, and the mirrored contralateral hemipelvis was used as the reduction template. After virtual reduction, the reduced position of the principal roof fragment was recorded. Three corresponding landmarks were then manually placed at reproducible positions on the fragment before and after reduction: two points along the main fracture line and one apex point of the fragment. The pre-reduction landmarks were defined as P1, P2, and P3, and the corresponding post-reduction landmarks were defined as P1′, P2′, and P3′. A plane was constructed using P1, P2, and P3, and a second plane was constructed using P1′, P2′, and P3′. Angular change was defined as the angle between these two planes. Supplementary Figure S1 illustrates the measurement workflow using a representative roof-column fragment as an example; the same procedure was applied to roof-wall fragments when present.

#### 2.8.3 Three-dimensional perifragment fracture-boundary length (mm)

This parameter was defined as the cumulative three-dimensional length of the complete fracture boundary surrounding the principal roof-related fragment after virtual reduction. After the principal fragment had been reduced to the hemipelvic template, the entire visible boundary between this fragment and the remaining pelvis was traced along the curved bone surface. The traced boundary included both the acetabular-side fracture margin and the extra-acetabular pelvic surface, including the medial iliac fossa region when involved. Therefore, the measured value represented the circumferential three-dimensional fracture-boundary length of the principal fragment, rather than a straight-line distance, a two-dimensional planar projection, or the length of a single fracture segment.

### 2.9 Reliability assessment

Two observers independently classified all 46 included cases into A3.1–A3.3 using blinded CT datasets. After a minimum 2-week washout, both observers repeated classification in a re-randomized order, blinded to prior results. Interobserver agreement was assessed using Cohen's kappa, and intraobserver agreement was assessed for each observer using Cohen's kappa.

For quantitative measurements (displacement, intra-/extra-fossa areas, angular change, and 3D perifragment fracture-boundary length), both observers performed measurements for all 46 cases. After a minimum 2-week washout, measurements were repeated in a re-randomized order with blinding to prior results. Interobserver reliability was assessed using ICC(2,1) (two-way random-effects, absolute agreement) based on Session 1 measurements. Intraobserver reliability was assessed using ICC(3,1) (two-way mixed-effects, absolute agreement) for Session 1 vs. Session 2 for each observer.

### 2.10 Statistical analysis

Categorical variables are presented as frequencies and percentages, and continuous variables as mean ± standard deviation (SD). Normality and homogeneity of variance were assessed before between-group comparisons. Continuous variables were compared among A3.1, A3.2, and A3.3 using one-way ANOVA when assumptions were met; Welch's ANOVA was used when homogeneity of variance was violated. Categorical variables were compared using Pearson's chi-square test without continuity correction as the primary test; Fisher's exact test was retained as a sensitivity analysis when expected cell counts were small. For continuous outcomes with significant overall between-group differences, *post-hoc* pairwise comparisons were performed using Welch's *t*-test with Bonferroni correction. Pairwise effect sizes were calculated as Hedges' g with 95% confidence intervals. Mean differences and Hedges' g were calculated as the first subtype minus the second subtype in each pairwise comparison. Omnibus effect sizes were calculated using eta-squared and omega-squared to quantify the magnitude of overall between-subtype differences. The 95% confidence intervals for eta-squared and omega-squared were estimated using bootstrap resampling. Statistical analyses were performed using SPSS 23.0 (IBM, NY, USA). A two-sided *P* value < 0.05 was considered statistically significant.

## 3 Results

### 3.1 Patient selection

**Figure 1:**
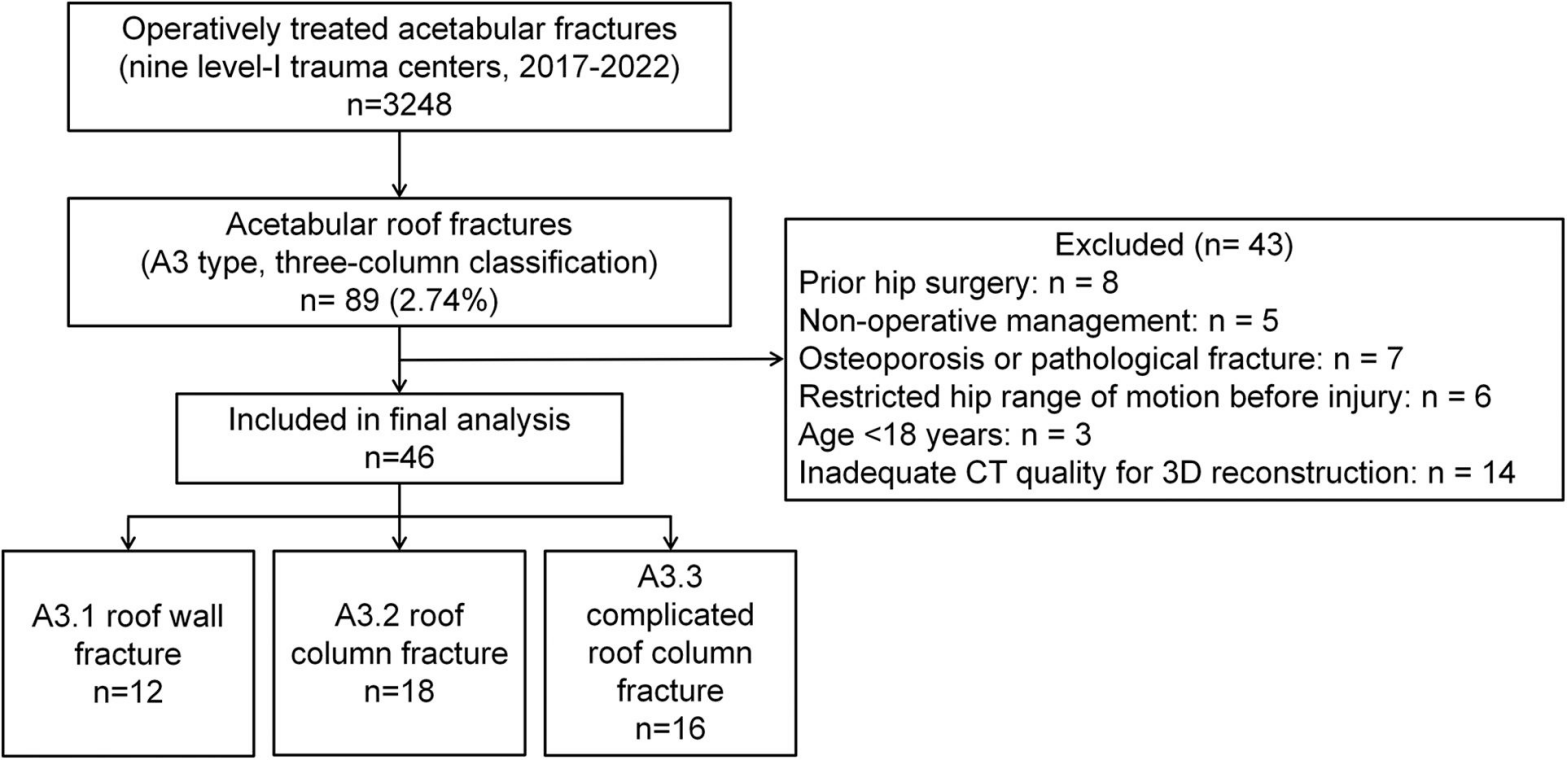
Flow diagram of patient selection. From January 2017 to December 2022, 3248 patients with acetabular fractures were screened at nine level-I trauma centers. Eighty-nine patients (2.74%) were classified as having A3 acetabular roof column or wall fractures. After exclusions (prior hip surgery, *n* = 8; non-operative management, *n* = 5; osteoporosis/pathological fracture, *n* = 7; restricted hip motion, *n* = 6; age <18 years, *n* = 3; inadequate CT quality, *n* = 14), no additional cases were excluded because of bilateral acetabular fractures or marked contralateral pelvic deformity. Finally, 46 surgically treated patients were included in the final analysis.

A total of 3,248 patients with acetabular fractures were screened at nine level-I trauma centers. Eighty-nine patients (2.74%) were identified as having A3 acetabular roof column or wall fractures. After exclusions (prior hip surgery, *n* = 8; non-operative management, *n* = 5; osteoporosis/pathological fracture, *n* = 7; restricted hip motion, *n* = 6; age <18 years, *n* = 3; inadequate CT quality, *n* = 14), no additional cases were excluded because of bilateral acetabular fractures or marked contralateral pelvic deformity. Finally, 46 surgically treated patients were included: A3.1 (*n* = 12), A3.2 (*n* = 18), and A3.3 (*n* = 16) (Figure 1).

#### 3.1.1 Baseline characteristics

The mean age was 44.93 ± 11.45 years, and 37 patients (80.4%) were male. Twenty-seven fractures (58.7%) involved the left side. Injury mechanisms were traffic accident (56.5%), fall from height (23.9%), slip/trip fall (13.0%), and crush injury (6.5%). High-energy mechanisms accounted for 87.0% of cases. Baseline characteristics did not differ significantly among subtypes (Table 1).

**Table 1:** Patient demographics and injury characteristics by A3 subtype.

Variable	Overall (*n* = 46)	A3.1 (*n* = 12)	A3.2 (*n* = 18)	A3.3 (*n* = 16)	*P* value
Age (years)	44.93 ± 11.45	43.67 ± 10.95	47.83 ± 12.37	42.62 ± 10.71	0.385
Male sex	37 (80.4)	9 (75.0)	13 (72.2)	15 (93.8)	0.247
Left side	27 (58.7)	10 (83.3)	10 (55.6)	7 (43.8)	0.103
Injury mechanism—traffic accident	26 (56.5)	9 (75.0)	10 (55.6)	7 (43.8)	0.565*
Injury mechanism—fall from height	11 (23.9)	2 (16.7)	3 (16.7)	6 (37.5)	
Injury mechanism—slip/trip fall	6 (13.0)	1 (8.3)	3 (16.7)	2 (12.5)	
Injury mechanism—crush injury	3 (6.5)	0 (0.0)	2 (11.1)	1 (6.2)	
High-energy injury	40 (87.0)	11 (91.7)	15 (83.3)	14 (87.5)	0.800

Values are presented as mean ± SD or *n* (%). *P* values compare A3.1, A3.2, and A3.3. One-way ANOVA or Welch's ANOVA was used for continuous variables, as appropriate. For categorical variables in Table 1, Pearson's chi-square test without continuity correction was used as the primary test; Fisher's exact test was retained as a sensitivity analysis when expected cell counts were small.

*For injury mechanism, the *P* value refers to the overall distribution across subtypes (chi-square test).

### 3.2 Reliability

For subtype classification, interobserver agreement was substantial (Session 1: κ = 0.767, 95% CI 0.601–0.902; Session 2: κ = 0.668, 95% CI 0.477–0.836). Intraobserver agreement was also substantial (Rater 1: κ = 0.734, 95% CI 0.557–0.896; Rater 2: κ = 0.700, 95% CI 0.508–0.864). Quantitative measurements showed excellent interobserver reliability [ICC(2,1) = 0.978–0.995] and excellent intraobserver reliability [ICC(3,1) = 0.979–0.997] (Supplementary Table S1).

### 3.3 Fracture mapping

**Figure 2:**
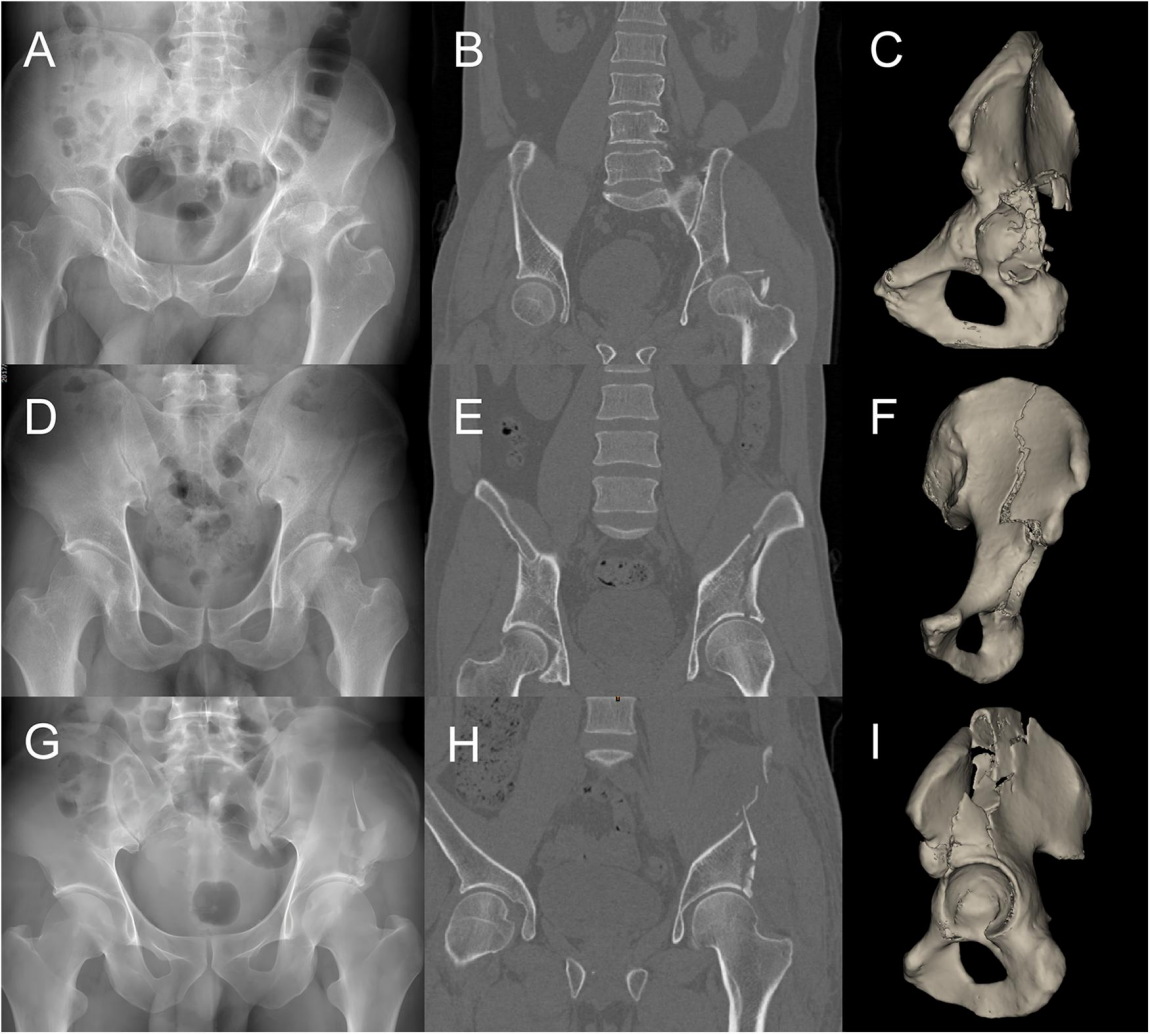
Representative radiographic, CT, and three-dimensional images of A3 acetabular roof injuries. **(A–C)** A3.1 roof wall fracture. **(D–F)** A3.2 roof column fracture. **(G–I)** A3.3 complex roof column fracture. For each subtype, the panels show an anteroposterior pelvic radiograph, a coronal CT image, and a three-dimensional reconstruction from one representative patient. Plain radiographs show suspected acetabular roof involvement, whereas coronal CT and three-dimensional reconstruction clarify the subtype-specific fracture morphology.

**Figure 3:**
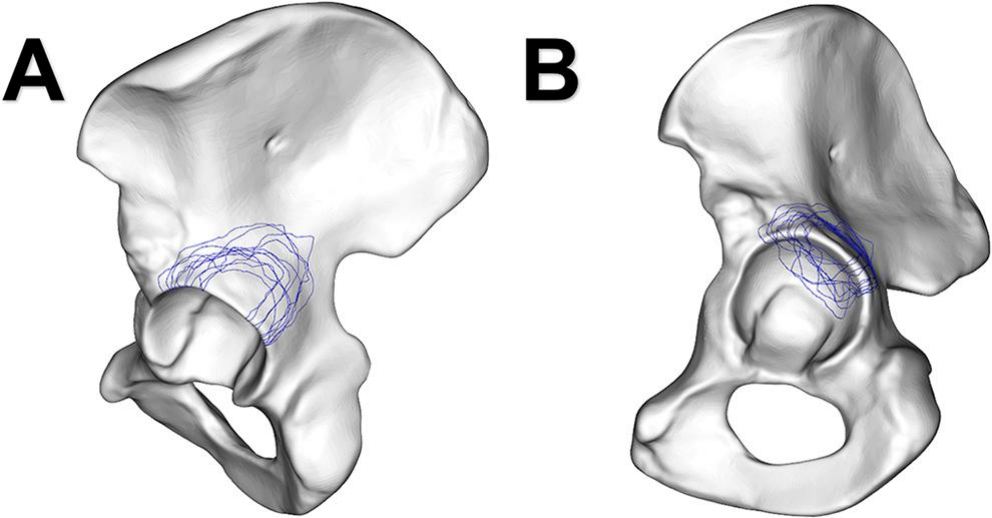
Three-dimensional fracture-line distribution of A3.1 roof wall fractures. **(A)** Oblique lateral view focused on the superior acetabular dome. **(B)** Oblique medial (endopelvic) view. After virtual reduction, fracture lines from 12 patients were traced on the reduced acetabular surface and superimposed on a standardized left hemipelvic template after scaling and rigid registration.

**Figure 4:**
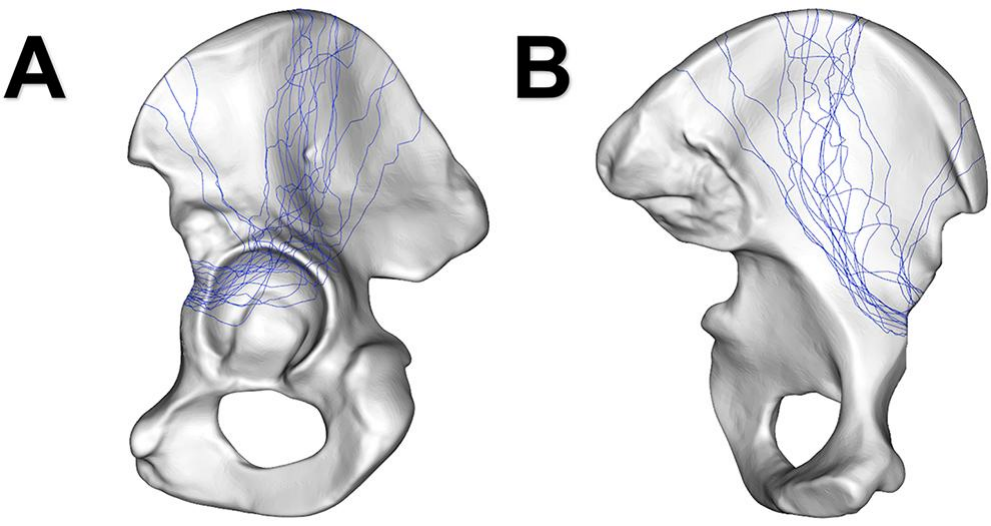
Three-dimensional fracture-line distribution of A3.2 roof column fractures. **(A)** Standard lateral view of the hemipelvis. **(B)** Standard medial (endopelvic) view. Superimposed fracture lines from 18 patients demonstrate a broader cranial extension toward the iliac crest compared to A3.1 injuries.

**Figure 5:**
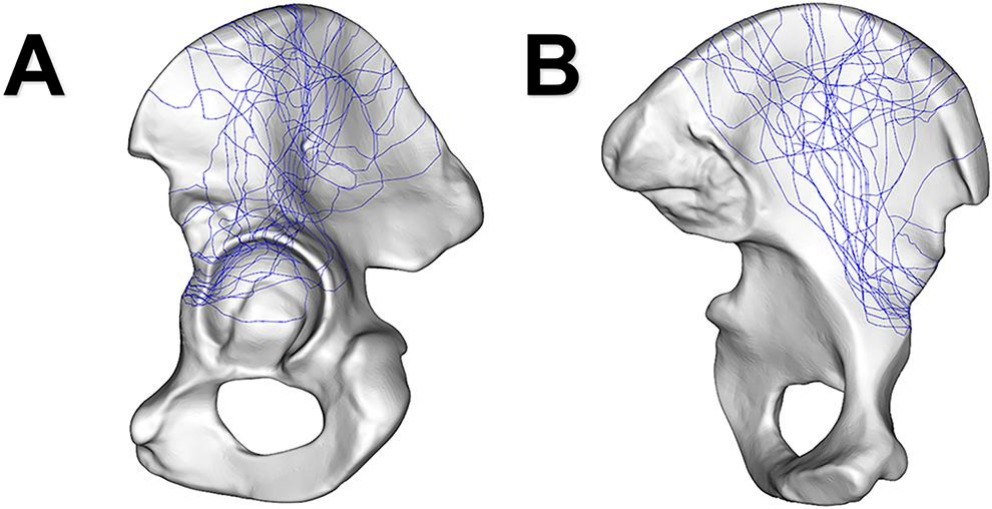
Three-dimensional fracture-line distribution of A3.3 complex roof column fractures. **(A)** Standard lateral view of the hemipelvis. **(B)** Standard medial (endopelvic) view. Superimposed fracture lines from 16 patients show extensive cranial propagation and a dispersed distribution pattern across both the roof and iliac wing.

**Figure 6:**
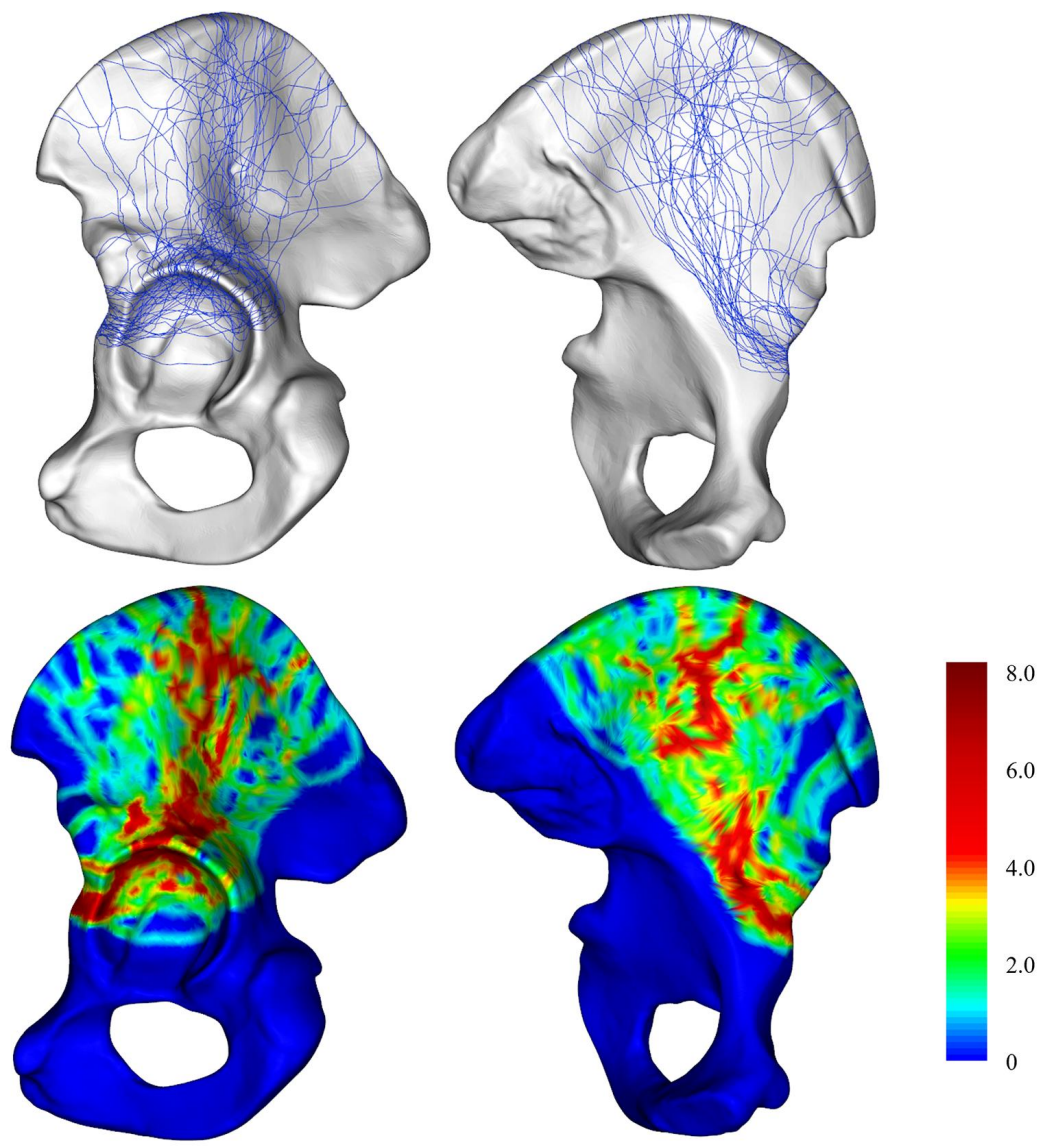
Three-dimensional fracture mapping and frequency heat map of A3 acetabular roof fractures. (Top row) Composite fracture maps displaying superimposed fracture lines (blue) from all 46 included patients on a standardized left hemipelvic template in standard lateral (left) and medial/endopelvic (right) views. (Bottom row) Corresponding frequency heat maps based on fracture-line density in both views. Warmer colors (towards red) indicate zones of higher fracture-line concentration. After virtual reduction, all fracture trajectories were scaled and rigidly registered to a standardized left-sided template, with right-sided cases mirrored to the left for overall standardization.

Representative anteroposterior pelvic radiographs, coronal CT images, and three-dimensional reconstructions of A3.1, A3.2, and A3.3 fractures are shown in Figure 2 to illustrate the radiologic appearance of each subtype. Subtype-specific superimposition suggested that A3.1 fracture lines (Figure 3) were relatively localized around the superior roof/dome region, whereas A3.2 and A3.3 (Figures 4, 5) showed broader cranial extension toward the iliac wing with a more dispersed distribution. Because of the limited number of fracture lines within each subtype, subtype-level frequency heat maps were not generated. In the overall cohort, the superimposed fracture map and frequency heat map (Figure 6) demonstrated that fracture lines clustered around the superior acetabular roof and frequently extended cranially into the adjacent iliac bone.

### 3.4 3D measurements

Overall mean 3D perifragment fracture-boundary length (measured after reduction) was 340.87 ± 105.74 mm. Because this metric represented the circumferential boundary length of the principal fragment across both acetabular and extra-acetabular pelvic surfaces, it should not be interpreted as the length of a single linear fracture line. Mean fragment displacement was 17.84 ± 9.14 mm. Mean intra-fossa surface area was 558.33 ± 343.91 mm^2^, and mean extra-fossa surface area was 4,200.87 ± 2,513.04 mm^2^. Mean angular change was 27.68 ± 19.53°.

Significant differences were found among subtypes for 3D perifragment fracture-boundary length (*P* < 0.001), fragment displacement (*P* = 0.003), extra-fossa area (*P* < 0.001), and angular change (*P* < 0.001) (Table 2). No statistically significant difference in intra-fossa surface area was detected among subtypes (*P* = 0.971). *Post-hoc* pairwise comparisons, including mean differences, Hedges' g values, 95% confidence intervals, and Bonferroni-adjusted *P* values, are provided in Supplementary Table S2. Omnibus effect sizes for the overall between-subtype comparisons are provided in Supplementary Table S3.

**Table 2:** Quantitative three-dimensional morphologic parameters of the main roof fragment by A3 subtype.

Variable	Overall (*n* = 46)	A3.1 (*n* = 12)	A3.2 (*n* = 18)	A3.3 (*n* = 16)	*P* value
3D perifragment fracture-boundary length (mm)	340.87 ± 105.74	183.68 ± 34.75	381.49 ± 63.19	413.06 ± 28.75	<0.001
Fragment displacement (mm)	17.84 ± 9.14	25.17 ± 9.12	15.28 ± 7.83	15.23 ± 7.89	0.003
Intra-fossa surface area (mm^2^)	558.33 ± 343.91	544.79 ± 313.77	573.84 ± 337.86	551.04 ± 390.86	0.971
Extra-fossa surface area (mm^2^)	4,200.87 ± 2,513.04	980.95 ± 366.72	4,949.39 ± 2,247.24	5,773.73 ± 1,232.33	<0.001
Angular change (°)	27.68 ± 19.53	51.90 ± 21.00	19.19 ± 10.53	19.07 ± 7.74	<0.001

Values are presented as mean ± SD. 3D perifragment fracture-boundary length was measured on the reduced model. Fragment displacement was the mean of three landmark-based Euclidean distances between pre-reduction and post-reduction fragment positions. Angular change was defined as the angle between the pre-reduction and post-reduction planes constructed from three corresponding landmarks on the main roof fragment. *P* values were calculated using one-way ANOVA when homogeneity of variance was met and Welch's ANOVA when homogeneity of variance was violated. Bonferroni-adjusted pairwise comparisons and pairwise effect sizes with 95% confidence intervals are provided in Supplementary Table S2. Omnibus effect sizes for between-subtype comparisons are provided in Supplementary Table S3.

## 4 Discussion

This multicenter 3D mapping study showed that A3 acetabular roof column/wall fractures include two different morphologic patterns rather than a single injury type. A3.1 fractures were relatively localized, with shorter post-reduction 3D perifragment fracture-boundary length, but they showed greater displacement and angular change. In contrast, A3.2 and A3.3 fractures had longer fracture trajectories and larger extra-fossa components. The relatively large numerical values for three-dimensional perifragment fracture-boundary length reflect the circumferential fracture boundary surrounding the principal roof-related fragment across the acetabular and extra-acetabular pelvic surfaces, rather than the length of a single fracture segment. No statistically significant difference in intra-fossa surface area was detected among subtypes, but this finding should not be interpreted as evidence that intra-fossa involvement is structurally equivalent across subtypes. Taken together, these findings suggest two morphology-based patterns: A3.1 injuries are characterized mainly by displacement and rotation of a relatively localized roof fragment, whereas A3.2 and A3.3 injuries show broader column-based cranial extension.

The clinical importance of these findings lies in the role of the acetabular roof as the superior weight-bearing area of the hip. Long-term studies and clinical reviews have consistently shown that articular congruity, femoral head containment, and the quality of reduction are major determinants of hip survival after acetabular fracture fixation (11, 12). The roof-arc concept was developed for the same reason: fractures involving the superior dome have greater implications for stability and post-traumatic arthritis than fractures outside the main weight-bearing zone (13, 14). Therefore, the morphologic differences observed in this study are not only descriptive. A localized roof fragment with marked rotation, as seen in A3.1 fractures, may still threaten joint congruity if derotation is incomplete. Conversely, the long cranial fracture course in A3.2 and A3.3 fractures may make maintenance of reduction dependent on stable control of the associated column component.

For A3.1 fractures, the greater angular change observed in this study suggests that rotational displacement of the roof fragment may be a key morphology-based concern. These injuries had the greatest angular change despite the shortest 3D perifragment fracture-boundary length. Thus, fragment size or fracture extent may underestimate the difficulty of reduction. These findings indicate that the rotational orientation of the roof fragment may warrant careful assessment on preoperative CT or three-dimensional reconstruction. From a morphology-based perspective, apparent restoration of the bony outline on standard views may not fully reflect rotational correction of the superior dome. Future studies incorporating intraoperative or postoperative CT assessment may help clarify whether rotational correction of the roof fragment is associated with reduction quality or clinical outcomes. This point is particularly relevant because even a small residual step or rotational incongruity in the weight-bearing dome may alter load transfer across the hip.

For A3.2 and A3.3 fractures, the problem is different. These subtypes had longer 3D perifragment fracture-boundary length and much larger extra-fossa surface areas, suggesting that the roof fragment is linked to a broader iliac or column segment. In these injuries, the broader cranial extension suggests that future biomechanical and clinical studies should evaluate the role of column control in maintaining reduction of the articular roof. A dome-focused reduction without adequate consideration of the associated column component may theoretically be vulnerable to loss of reduction, particularly when the extra-fossa component is large. This morphology provides a rationale for future studies evaluating whether broader implant coverage, column-based buttressing, or multi-planar fixation improves reduction maintenance or clinical outcomes. The present data do not prove that one fixation construct is superior to another; rather, they suggest that A3.2 and A3.3 fractures have morphologic features that may warrant further investigation in relation to fixation coverage and reduction maintenance.

The mapping results may provide an anatomical reference for considering exposure-related questions, although they do not allow direct comparison of surgical approaches. Modern intrapelvic, modified Stoppa, and pararectus approaches have been used to improve access to the quadrilateral surface, anterior column, and superior acetabular region, and comparative studies have reported their perioperative features and reduction outcomes (15–19). The modified Stoppa approach is an anterior intrapelvic approach performed through a lower midline incision, allowing exposure of the pelvic brim, quadrilateral surface, and medial acetabular structures. For A3 injuries, the dominant morphologic pattern may be considered during preoperative assessment, although the present study does not directly compare surgical approaches. In A3.1 fractures, the greater angular change observed in this study suggests that derotation of the dome-adjacent fragment may be an important morphology-based consideration. In A3.2 and A3.3 fractures, the broader cranial extension and larger extra-fossa component may be relevant when future studies evaluate exposure requirements and implant coverage. Adjuncts such as infra-acetabular screws, supra-acetabular fixation, or navigation-assisted screw placement may be considered in future studies of column linkage and screw safety, but the present data cannot determine their indications or comparative effectiveness (20, 21).

The present findings also emphasize the complementary roles of plain radiography and CT-based three-dimensional assessment. Plain anteroposterior pelvic radiographs and Judet views remain useful for initial screening and for identifying suspected acetabular roof involvement. However, overlapping pelvic structures may limit their ability to define roof-wall vs. roof-column morphology, cranial fracture extension, fragment rotation, and small dome-adjacent fragments. Thin-slice CT with multiplanar reconstruction provides more detailed assessment of the articular surface and fracture trajectory, while three-dimensional reconstruction further improves visualization of spatial relationships and allows standardized mapping and quantitative measurements. Because this study was not designed as a diagnostic accuracy study, we did not calculate the sensitivity or specificity of plain radiography vs. CT or three-dimensional reconstruction for A3 subtype classification. Future studies should evaluate the diagnostic performance of different imaging modalities for identifying A3.1, A3.2, and A3.3 injuries. Another value of this study is that it adds quantitative information to conventional fracture classification. Classification systems are necessary for communication, but they do not directly measure clinically relevant morphologic features, including displacement, rotation, surface involvement, and fracture extent. Previous 3D CT-based studies have shown that acetabular fracture lines and comminution zones follow reproducible patterns, supporting the use of fracture mapping for preoperative assessment (22). Patient-specific 3D planning and 3D printing may also improve preoperative understanding in complex pelvic and acetabular trauma (23, 24). In the present study, the quantitative measurements showed excellent reliability, suggesting that these parameters can be measured reproducibly. This is important because radiographs and fluoroscopy may underestimate residual gaps or step-offs, whereas CT-based assessment is more sensitive for postoperative reduction evaluation and has been associated with hip survivorship (25, 26).

The absence of a statistically significant difference in intra-fossa surface area among subtypes should be interpreted cautiously. The acetabular fossa is not the same as the superior weight-bearing dome, and the absence of a statistically significant difference in fossa surface area does not imply equivalent clinical risk. For roof injuries, the more important issue may be how the fragment is oriented relative to the dome and how far the fracture extends into the supporting column. In addition, this study did not quantify marginal impaction, dome impaction, or osteochondral depression. Previous studies have shown that marginal or osteochondral impaction can compromise joint congruity and prognosis if it is not recognized and anatomically restored (27, 28). Recent reviews also emphasize the need for thin-slice CT to detect articular impaction and to guide fragment-specific elevation, grafting, or subchondral support when needed (29). Future studies of A3 fractures should therefore combine the present 3D parameters with measurements of dome impaction, comminution, step-off, and femoral head injury.

This study has several limitations. First, it was retrospective and included only surgically treated patients. Therefore, the fracture maps likely represent the more displaced, unstable, or complex end of the A3 fracture spectrum, and the findings may not generalize to minimally displaced or stable A3 injuries managed nonoperatively. Second, the subgroup sizes were small, which limited statistical power and increased the risk of Type II error. In particular, the absence of a statistically significant difference in intra-fossa surface area should not be interpreted as evidence of structural equivalence among subtypes. No formal *post-hoc* power analysis was performed. Third, CT acquisition parameters varied across centers, which may have influenced segmentation and mapping accuracy. Fourth, selection of the principal roof fragment may introduce measurement bias in multifragmentary fractures, although borderline cases were reviewed by two observers and adjudicated by a senior acetabular surgeon. Fifth, although angular measurements showed excellent interobserver and intraobserver reliability, the sensitivity of angular change to small landmark-placement errors was not formally tested. Sixth, the present workflow focused on cortical fracture-boundary distribution and the principal roof fragment. Marginal impaction, dome impaction, osteochondral depression, cartilage injury, and femoral head damage were not quantified separately, although these features are clinically important determinants of reduction quality and prognosis. Finally, surgical approach, implant configuration, postoperative reduction quality, complications, and functional outcomes were not analyzed. Therefore, the surgical implications discussed above should be considered morphology-based hypotheses rather than treatment recommendations. Larger studies should determine whether displacement, angular change, extra-fossa area, or three-dimensional perifragment fracture-boundary length predicts reduction difficulty, fixation failure, post-traumatic arthritis, or conversion to total hip arthroplasty. Standardized outcome reporting will also be important for comparing future studies of acetabular fracture morphology and treatment (30).

In summary, this study identified two morphology-based patterns among A3 acetabular roof injuries. A3.1 fractures were relatively localized but showed greater displacement and angular change, highlighting roof-fragment orientation as a key morphology-based concern. In contrast, A3.2 and A3.3 fractures showed broader cranial extension, larger extra-fossa components, and greater three-dimensional perifragment fracture-boundary length. These findings provide an anatomical basis for future studies evaluating the relationship between subtype-specific fracture morphology, reduction quality, fixation coverage, and clinical outcomes.

## 5 Conclusion

Quantitative 3D assessment suggests that A3 acetabular roof injuries include distinct morphology-based patterns. Roof-wall fractures were relatively localized but showed greater displacement and angular change, indicating that rotational malreduction of the roof fragment may be a key concern in A3.1 injuries. Roof-column and complex roof-column fractures showed broader extra-fossa involvement and greater three-dimensional perifragment fracture-boundary length, suggesting more extensive cranial extension in A3.2 and A3.3 injuries. These subtype-specific morphologies may help refine morphology-informed preoperative assessment and provide hypotheses for future evaluation of reduction and fixation strategies. Because this cohort was restricted to surgically treated patients with adequate CT data, generalization to minimally displaced or nonoperatively managed A3 injuries should be cautious. However, these implications should be interpreted as morphology-based hypotheses and require validation in future studies incorporating reduction quality, fixation constructs, and clinical outcomes.

## Data Availability

The datasets presented in this article are not readily available because the original contributions presented in the study are included in the article and Supplementary Material. The patient-level clinical and imaging-derived dataset generated and analyzed during the current study is not publicly available because of patient privacy and ethical restrictions. Requests to access the datasets should be directed to Ruipeng Zhang (38900330@hebmu.edu.cn), subject to approval by the Ethics Committee of the Third Hospital of Hebei Medical University. Requests to access the datasets should be directed to 38900330@hebmu.edu.cn.

## Supplementary material

Supplementary Material The supplementary material for this article can be found online. Supplementary Figure S1 illustrates the angular change measurement workflow. Supplementary Table S1 presents the reliability analysis for fracture classification and quantitative measurements. Supplementary Table S2 presents the Bonferroni-adjusted pairwise comparisons, mean differences, Hedges' g values, and 95% confidence intervals for quantitative three-dimensional morphologic parameters among A3 subtypes. Supplementary Table S3 presents omnibus effect sizes for subtype-specific morphologic comparisons.
