# Supplementary material for: Quantitative three-dimensional fracture mapping reveals subtype-specific morphology of acetabular roof column and wall fractures
Source: Front Surg. 2026 Jun 29;13:1879706. doi: 10.3389/fsurg.2026.1879706 (PMC13357869; doi:10.3389/fsurg.2026.1879706)
Supplement: Supplementary Material — The supplementary material for this article can be found online. Supplementary Figure S1 illustrates the angular change measurement workflow. Supplementary Table S1 presents the reliability analysis for fracture classification and quantitative measurements. Supplementary Table S2 presents the Bonferroni-adjusted pairwise comparisons, mean differences, Hedges' g values, and 95% confidence intervals for quantitative three-dimensional morphologic parameters among A3 subtypes. Supplementary Table S3 presents omnibus effect sizes for subtype-specific morphologic comparisons. [file Datasheet1.docx]

Supplementary Material

# Supplementary Figures

**Supplementary Figure S1. Workflow for measuring angular change of the main roof fragment.**


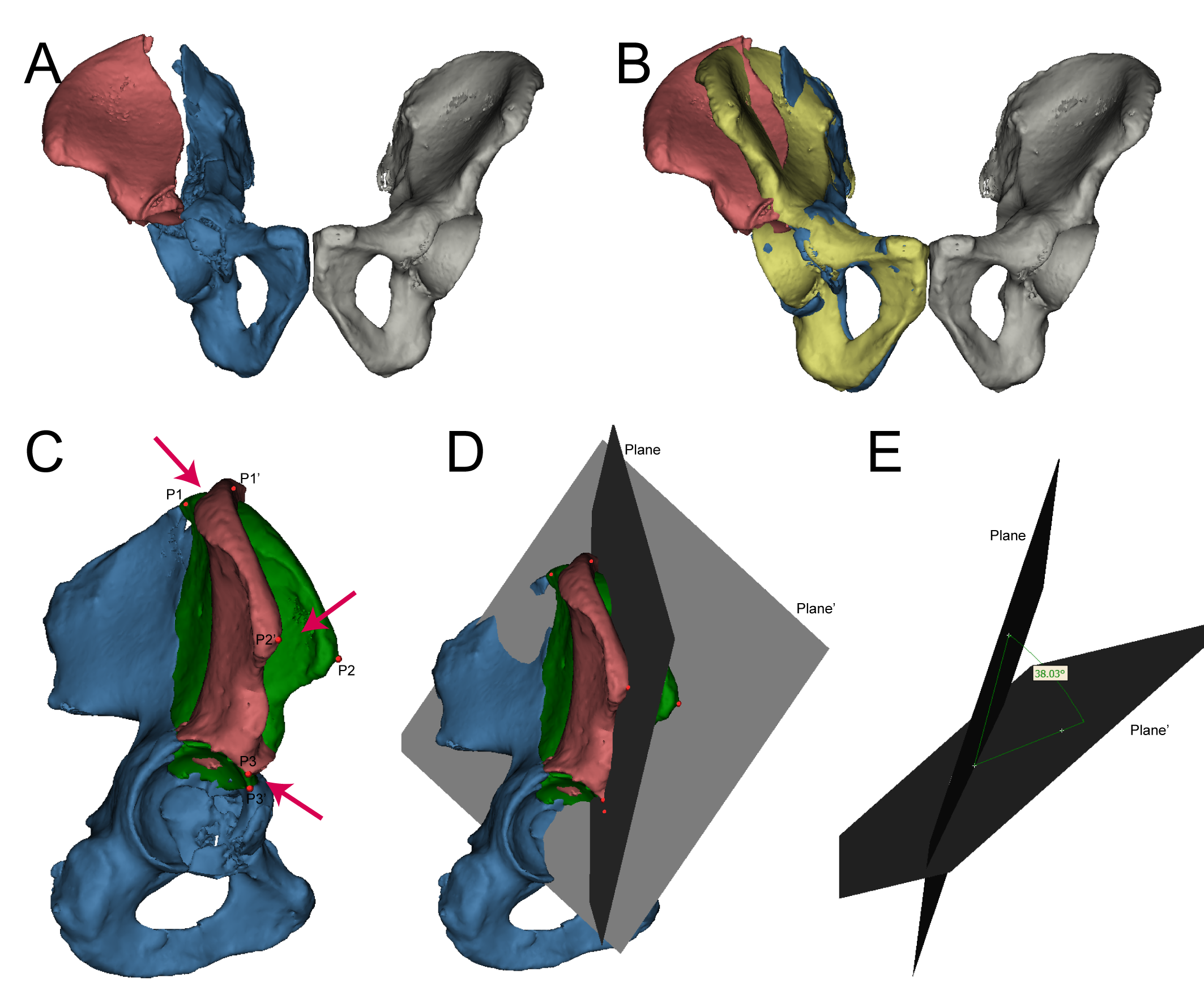
(A) Three-dimensional reconstruction of the injured hemipelvis with fracture fragments segmented in different colors. The reddish fragment represents the principal roof fragment. (B) The mirrored contralateral hemipelvis, shown in yellow, was used as the template for virtual reduction of the injured side. (C) After virtual reduction, the reduced principal roof fragment is shown in green. Three corresponding landmarks were manually placed on the fragment before and after reduction: P1 and P1′, P2 and P2′, and P3 and P3′. (D) A pre-reduction plane was constructed using P1, P2, and P3, and a post-reduction plane was constructed using P1′, P2′, and P3′. (E) Angular change was defined as the angle between the pre-reduction and post-reduction planes, representing rotational displacement of the principal roof fragment. The angle value shown is from a representative case.

# Supplementary Tables

**Supplementary Table S1. Reliability of classification and quantitative measurements**

**Table A. Classification reliability (Cohen’s kappa)**

| Comparison | Percent agreement | Kappa (κ) | 95% CI |
| --- | --- | --- | --- |
| Interobserver (Rater1 vs Rater2, Session 1) | 84.8% | 0.767 | 0.601–0.902 |
| Interobserver (Rater1 vs Rater2, Session 2) | 78.3% | 0.668 | 0.477–0.836 |
| Intraobserver (Rater1, Session 1 vs 2) | 82.6% | 0.734 | 0.557–0.896 |
| Intraobserver (Rater2, Session 1 vs 2) | 80.4% | 0.700 | 0.508–0.864 |

**Table B. Measurement reliability (Intraclass correlation coefficients)**

| Metric | Interobserver ICC(2,1) (95% CI)* | Intraobserver ICC(3,1) Rater1 (95% CI)* | Intraobserver ICC(3,1) Rater2 (95% CI)* |
| --- | --- | --- | --- |
| Fragment displacement (mm) | 0.978 (0.962–0.985) | 0.979 (0.962–0.989) | 0.982 (0.965–0.991) |
| Intra-fossa surface area (mm²) | 0.994 (0.991–0.996) | 0.996 (0.991–0.998) | 0.996 (0.992–0.998) |
| Extra-fossa surface area (mm²) | 0.995 (0.993–0.997) | 0.997 (0.995–0.998) | 0.996 (0.993–0.998) |
| Angular change (°) | 0.989 (0.980–0.993) | 0.993 (0.986–0.996) | 0.991 (0.985–0.994) |
| 3D perifragment fracture-boundary length (mm) | 0.980 (0.964–0.987) | 0.992 (0.985–0.995) | 0.994 (0.990–0.996) |

Notes: Interobserver ICC(2,1) was calculated using Session 1 measurements with a two-way random-effects model and absolute agreement. Intraobserver ICC(3,1) was calculated for Session 1 vs Session 2 for each rater using a two-way mixed-effects model and absolute agreement.
*95% CIs were calculated based on the corresponding kappa and ICC models.

**Supplementary Table S2. Pairwise comparisons of quantitative three-dimensional morphologic parameters among A3 subtypes**

| Quantitative parameter | Comparison | Mean difference (95% CI) | Hedges’ g (95% CI) | Bonferroni-adjusted P value |
| --- | --- | --- | --- | --- |
| 3D perifragment fracture-boundary length (mm) | A3.1 vs A3.2 | -197.81 (-234.65 to -160.98) | -3.57 (-4.76 to -2.39) | <0.001 |
| 3D perifragment fracture-boundary length (mm) | A3.1 vs A3.3 | -229.39 (-255.04 to -203.73) | -7.09 (-9.15 to -5.02) | <0.001 |
| 3D perifragment fracture-boundary length (mm) | A3.2 vs A3.3 | -31.57 (-65.68 to 2.54) | -0.62 (-1.31 to 0.07) | 0.204 |
| Fragment displacement (mm) | A3.1 vs A3.2 | 9.89 (3.21 to 16.58) | 1.15 (0.36 to 1.94) | 0.017 |
| Fragment displacement (mm) | A3.1 vs A3.3 | 9.94 (3.12 to 16.77) | 1.14 (0.33 to 1.96) | 0.019 |
| Fragment displacement (mm) | A3.2 vs A3.3 | 0.05 (-5.46 to 5.55) | 0.01 (-0.67 to 0.68) | 1.000 |
| Extra-fossa surface area (mm²) | A3.1 vs A3.2 | -3968.44 (-5101.76 to -2835.12) | -2.19 (-3.11 to -1.26) | <0.001 |
| Extra-fossa surface area (mm²) | A3.1 vs A3.3 | -4792.79 (-5476.12 to -4109.45) | -4.82 (-6.33 to -3.31) | <0.001 |
| Extra-fossa surface area (mm²) | A3.2 vs A3.3 | -824.34 (-2081.73 to 433.04) | -0.44 (-1.12 to 0.25) | 0.569 |
| Angular change (degrees) | A3.1 vs A3.2 | 32.71 (18.73 to 46.70) | 2.05 (1.15 to 2.96) | <0.001 |
| Angular change (degrees) | A3.1 vs A3.3 | 32.83 (19.10 to 46.55) | 2.14 (1.19 to 3.09) | <0.001 |
| Angular change (degrees) | A3.2 vs A3.3 | 0.11 (-6.31 to 6.53) | 0.01 (-0.66 to 0.69) | 1.000 |

Notes: CI, confidence interval. Mean difference and Hedges’ g were calculated as the first subtype minus the second subtype in each comparison. Pairwise comparisons were performed using Welch’s t test with Bonferroni correction. Post-hoc pairwise comparisons are presented only for quantitative parameters with significant overall between-subtype differences in Table 2.

**Supplementary Table S3. Omnibus effect sizes for subtype-specific morphologic comparisons**

| Quantitative parameter | Overall P value | Eta-squared (95% CI) | Omega-squared (95% CI) |
| --- | --- | --- | --- |
| 3D perifragment fracture-boundary length (mm) | <0.001 | 0.814 (0.672 to 0.930) | 0.802 (0.651 to 0.925) |
| Fragment displacement (mm) | 0.003 | 0.232 (0.070 to 0.478) | 0.193 (0.026 to 0.448) |
| Intra-fossa surface area (mm²) | 0.971 | 0.001 (0.001 to 0.163) | 0.000 (0.000 to 0.121) |
| Extra-fossa surface area (mm²) | <0.001 | 0.613 (0.432 to 0.811) | 0.589 (0.400 to 0.798) |
| Angular change (degrees) | <0.001 | 0.555 (0.339 to 0.754) | 0.529 (0.304 to 0.738) |

Notes: CI, confidence interval. Overall P values correspond to the between-subtype comparisons reported in Table 2. Eta-squared and omega-squared were calculated to quantify the magnitude of between-subtype differences; 95% confidence intervals were estimated using bootstrap resampling.
